# A novel approach for finding ring species: look for barriers rather than rings

**DOI:** 10.1186/1741-7007-10-21

**Published:** 2012-03-12

**Authors:** Darren E Irwin

**Affiliations:** 1 Biodiversity Research Centre and Department of Zoology, University of British Columbia, 6270 University Blvd, Vancouver, BC, V6T 1Z4, Canada

## Abstract

Ring species, in which two different forms coexist in one region while being connected by a long chain of interbreeding populations encircling a geographic barrier, provide clear demonstrations of the evolution of one species into two. Known ring species are rare, but now Monahan *et al*. propose an intriguing new approach to discovering them: focus first on geography to find potential barriers.

See research article http://www.biomedcentral.com/1741-7007/10/20

## Commentary

**Figure 1:**
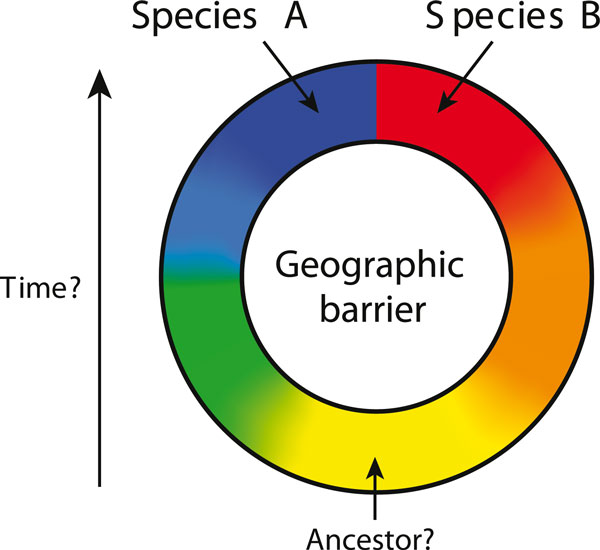
**Map of the geographic distribution of an idealized ring species**. Two forms (red and blue; species A and B) have come into contact (perhaps with some overlap) but do not interbreed directly. They are connected by a long chain of populations encircling a geographic barrier, through which the traits of species A gradually change into the traits of species B. If the order of colonization can be inferred, then one can infer the location of the common ancestor (here, in yellow) and how range expansion around the barrier and the accumulation of small evolutionary changes led to the formation of two species.

Speciation, the process by which a single species evolves into two or more, is difficult to observe directly because of the long span of time it usually takes to occur. Nonetheless, biologists have been able to infer much about speciation by examining geographic variation within and between species. A striking pattern that emerged about a century ago is known as Jordan's law [1]: given any species, the most closely related species is found 'in a neighboring district separated from the first by a barrier of some sort or at least by a belt of country, the breadth of which gives the effect of a barrier.' The role of such barriers in speciation is perhaps best illustrated by the rare phenomenon known as 'circular overlaps' [2] or 'ring species' [3], when two coexisting but reproductively isolated forms are connected by a long chain of populations encircling a geographic barrier, and traits change gradually from those of one form to the other around the ring [4] (Figure 1). The great evolutionary biologist Ernst Mayr called such situations the 'perfect demonstration of speciation' [2] since they allow one to use geographic variation to infer how evolutionary change in time led to the differences between species.

Until now, our knowledge of the diversity of ring species has arisen primarily from the field of taxonomy, with experts on the taxonomy of particular groups occasionally noticing a pattern of gradual variation between quite divergent forms. This somewhat haphazard approach has led to a variety of ring species being proposed [2,4], only some of which have held up to further scrutiny [4,5]. Only two well-studied cases are generally accepted as solid examples of ring species: these are the *Ensatina eschscholtzii *salamander complex in California [6] and the *Phylloscopus trochiloides *greenish warbler complex in Asia [7]. One challenge in relying on taxonomists to discover ring species is that the naming rules of taxonomy generally conceal their existence: taxonomists have to decide whether a group of specimens is two species or one species; the taxonomic naming system does not lend itself toward describing gradients between two species [4].

The study by Monahan *et al*. [8] proposes a novel approach to the discovery of ring species, focusing on geography rather than taxonomy as the starting point. They ask an intriguing question: where in the world are there barriers that might promote ring speciation? A topographic model, based on slope of the landscape, is used to identify potential geographic barriers worldwide. In the model, barriers are regions that have either more or less slope than the regions around them. The characteristics of the potential barriers, such as size and shape, are then compared with those of known barriers in two ring species (*E. eschscholtzii *salamanders and *P. trochiloides *greenish warblers) and two groups that have been proposed as ring species and share many of their characteristics (*Acacia karoo *trees and *Larus *gulls). Known barriers are similar to only a small proportion of all potential barriers, suggesting that ring species barriers have common characteristics. The authors also show maps of a small subset of the potential barriers that are similar to the real ring species barriers, suggesting that these may be good locations to look for ring species.

Though the current model is based solely on slope, other geographic and environmental variables could eventually be incorporated to enhance the effectiveness of the model in identifying some barriers in species distributions. In particular, it may be advantageous to introduce elevation as a geographic variable in the model. The current use of slope results in two sorts of 'barriers' being identified: 1) areas of high slope, such as mountain ranges, escarpments, or ocean trenches, surrounded by areas of low slope such as plains, plateaus, or ocean basins; and 2) areas of low slope surrounded by those of high slope. As a result, some of the barriers identified by this model are peculiar: for example, in the first case, an area of flat land bordered on one side by a steep climb toward higher elevations and on the other side by a steep drop toward lower elevations; in the second case, a steep escarpment between a high plateau and a low plain. In both of these, it seems unlikely that a species could live in all areas encircling the 'barrier' without also inhabiting the 'barrier' itself. Rather, it seems that the optimal topographic model would use some combination of both slope and elevation to identify barriers. Elevation is also likely to work better than slope in describing the Arctic Ocean barrier in the case of the *Larus *gull ring; the slope-based model results in three separate barriers corresponding to deep ocean basins, which the authors then joined as a composite barrier (see [8], their Figure 2D). It seems that slope on the deep ocean floor is of little relevance to describing the distribution of a bird species, whereas elevation (for example, above or below sea level) is of substantial importance.

Environmental variables such as climate or vegetation could also be incorporated into the model. For instance, with respect to the central Asian barrier that the greenish warbler encircles, Monahan *et al*. find that their model did not identify a single barrier - rather, they construct a composite barrier out of two separate barriers identified by the model. They remark that, in cases such as this, 'it is difficult to imagine any univariate or multivariate environmental approximation of a single barrier (for example, Central Asia, which is comprised of the Takla Maka-Gobi deserts and the Tibetan Plateau - large geographic regions that differ dramatically in terms of climate and vegetation).' However, a good explanatory variable has been identified in this case: greenish warblers inhabit forests [7], and maps of forests in Asia (for example, [9]) show a large gap that includes the Tibetan Plateau as well as the Taklamakan and Gobi deserts. Other examples of large potential barriers that show up clearly when considering a basic environmental variable (wet versus dry) are Antarctica, Australia, and Greenland (for marine and/or terrestrial coastal organisms), which were missed by the current topographic model. It is clear that the addition of other topographic and environmental variables could greatly enhance the precision of the model, and Monahan *et al*. [8] emphasize that their general approach can be modified to work with any kind of continuously distributed environmental variable, making it of wide applicability to many different types of investigations into barriers to dispersal that may contribute to speciation.

Finally, the very large number of potential barriers identified by the topographic model (952,147, about 10,000 of which are 'topographically similar' to those associated with known ring taxa [8]) raises another issue. Given the very large number of identified candidate barriers, it is almost inevitable that at least one will be associated with any interesting species complex that we might point to as a candidate for ring speciation, and this means that the predictive value of the model will depend on further refinement. Despite these issues, it is likely that the present model represents an important first step in this geography-oriented approach to the analysis of barriers involved in both ring speciation and speciation more generally. The approach proposed by Monahan *et al*. [8] will likely be adapted to incorporate multiple variables (in addition to slopes), and this will allow more refined identifications of a smaller number of potential barriers, resulting in more useful predictions. The discovery and inclusion of more ring species (for example, the willow warblers *Phylloscopus trochilus*, which display a form of incipient ring speciation around the Baltic Sea [5,10]) will likewise allow further refinement of the model, perhaps eventually allowing an analysis of what types of barriers are associated with ring species from different taxonomic groups. By applying an explicit geographic framework to the analysis of ring species, Monahan *et al*. have pioneered an interesting new approach to the study of the relationship between geography and speciation. In the years ahead, it will be exciting to see whether additional ring species are identified using this geography-oriented approach.
